# Clinical and Pathological Reports of Cases of Insanity

**Published:** 1884-08

**Authors:** S. V. Clevenger

**Affiliations:** Special Pathologist Cook County Insane Asylum; Chicago


### ARTICLE III.

CLINICAL AND PATHOLOGICAL REPORTS OF CASES OF INSANITY. *By* S. V. CLEVENGER, M.D., *Special Pathologist Cook County Insane Asylum.*

| | Males. | Females. | Total. |
|---|---|---|---|
| Present June 1, 1883 | 227 | 273 | 500 |
| Admitted during year | 224 | 223 | 447 |
| Total under observation | 451 | 496 | 947 |
| Of whom died | 56 | 66 | 122 |
| Of whom were discharged | 86 | 95 | 181 |
| Present June 1, 1884 | 309 | 335 | 644 |

From October to February the death rate was less than during the other parts of the year.

The Psychoses of those admitted during the year ending June 1, 1884, were as follows:

| | Males. | Females. | Total. |
|---|---|---|---|
| Alcoholic Insanity | 34 | 9 | 43 |
| Circular " | 2 | | 2 |
| Epileptic " | 15 | 12 | 27 |
| Hebephrenia | 7 | 4 | 11 |
| Hysterical Insanity | | 8 | 8 |
| Hypomania | | 1 | 1 |
| Imbecility | 3 | 3 | 6 |
| Katatonia | 5 | 2 | 7 |
| Mania | 54 | 49 | 103 |
| Melancholia | 42 | 59 | 101 |
| Paranoia | 7 | 9 | 16 |
| Paretic Dementia | 11 | 2 | 13 |
| Primary Confusional Insanity | 4 | 6 | 10 |
| Recurrent Alcoholic | 1 | | 1 |
| " Mania | 6 | 8 | 14 |

| | Males. | Females. | Total. |
|---|---|---|---|
| Recurrent Melancholia.......................... | 2 | 5 | 7 |
| Rheumatic Insanity................................ | 1 | 1 | 2 |
| Secondary Confusional Insanity.............. | 2 | 4 | 6 |
| Senile Dementia.................................... | 7 | 18 | 25 |
| Stuporous Insanity................................ | 5 | 1 | 6 |
| Syphilitic " ................................ | 2 | 1 | 3 |
| Traumatic " ................................ | 4 | | 4 |
| Transitory Frenzy................................ | | 1 | 1 |
| Terminal Dementia................................ | 10 | 15 | 25 |
| Typhomania........................................ | | 2 | 2 |
| Undiagnosed. No Psychosis apparent..... | | 3 | 3 |
| | 224 | 223 | 447 |

One of the Paretics admitted was a negro.

The greater number of those discharged were upon admission maniacal, melancholiac or alcoholic, 28 of the first had been detained from 3 months to 3 years; 16 of them were discharged within 3 months of their admission.

Of the melancholiac, males, 19; females, 27; one-half remained less than 6 months, the others various periods, up to 2 years.

The alcoholics recovered most rapidly, often within two weeks. They also die soonest of any of the forms of insanity.

The discharges from among those admitted during the year were as follows:

| | Under 3 mos. | | Under 6 mos. | | Under 1 year. | |
|---|---|---|---|---|---|---|
| | M. | F. | M. | F. | M. | F. |
| Alcoholic Insanity............ | 9 | 4 | | 1 | | |
| Epileptic " ............ | 3 | 1 | 2 | 1 | | |
| Hebephrenia...................... | | | 2 | | 1 | |
| Hysterical Insanity........... | | 3 | | 1 | | 1 |
| Imbecility........................ | | 2 | | | | |

| | Under 3 mos. M. | Under 3 mos. F. | Under 6 mos. M. | Under 6 mos. F. | Under 1 year. M. | Under 1 year. F. |
|---|---|---|---|---|---|---|
| Mania | 10 | 9 | 1 | 2 | 1 | |
| Melancholia | 3 | 10 | 10 | 3 | 1 | 1 |
| Paranoia | 1 | 2 | 1 | 3 | | |
| Paretic Dementia | | | 1 | | | |
| PrimaryConfusional Insanity | 2 | 2 | | | | |
| Recurrent Mania | | 2 | 2 | 1 | | |
| " Melancholia | | 1 | | 2 | | 1 |
| " Alcoholic | | | | | 1 | |
| Rheumatic Insanity | | 1 | | | | |
| Senile Dementia | | 3 | | | | |
| Stuporous Insanity | | 1 | | | 1 | |
| Terminal Dementia | 1 | 1 | | 1 | | |
| Totals | 29 | 42 | 19 | 15 | 5 | 3 |

Total males, 53; females, 60.

The Terminal and Senile Dements, Imbeciles and Epileptics were merely taken home by their friends, unimproved. The Alcoholics made good recoveries, but there is no predicting when they would relapse through a return to their bad habits. I believe that the poisonous adulteration in liquors complicate this form of insanity, which is a true toxæmia in the beginning, and an organic disease later.

The hysterical for the most part never fully recover. They are inherently defective, but discipline will benefit them to a greater or less extent.

Mania and Melancholia are more hopeful forms, and doubtless one-half of those discharged may never have recurrences.

Paranoia is balanced between imbecility and hysteria, so far as recovery is concerned. A few cases under proper circumstances never relapse.

Paretic Dementia is, so far, absolutely hopeless, though I

believe very much more could be done for this kind of lunacy than has been thought possible in the past. During remissions paretics are often discharged.

The Primary Confusionals make the best recoveries and the most sudden. The stuporous slowly emerge, but in my opinion the predisposition to the latter form is in an abnormality of the circulation.

The Rheumatic and Syphilitic forms in their incipiency are the most curable psychoses.

The deaths this year among those who had been admitted before June 1, 1883, were mainly Terminal Dements, who among the males were maniacs and among the females melancholiacs after a residence in the asylum varying from a month to six years.

There are few differences in the ages to which the chronic insane live as they pass toward terminal dementia, but the paretics seldom pass the third year of their asylum residence alive. We have two paretics who have lived here over four years, and both are cases which began with locomotor ataxia. I have my doubts as to this so-called ascending paresis being correctly named. There are many differences between the insanity associated with this neurosis and the ordinary paretic dementia. Other asylum offlcials would do a service to medicine if they would note such cases apart, especially with reference to their differing in length of life from the genuine form.

Those who died from among the patients admitted after June 1, 1883, survived after admission as follows:

| | Under 3 mos. | | Under 6 mos. | | Under 1 year. | |
|---|---|---|---|---|---|---|
| | M. | F. | M. | F. | M. | F. |
| Alcoholic Insanity | 6 | | | | | |
| Circular " | 1 | | | | | |
| Epileptic " | | 1 | | | 1 | |
| Mania | 5 | 3 | | | 1 | |

| | Under 3 mos. | | Under 6 mos. | | Under 1 year. | |
|---|---|---|---|---|---|---|
| | M. | F. | M. | F. | M. | F. |
| Melancholia | | 9 | | 2 | | 2 |
| Paranoia | | | | | | 1 |
| Paretic Dementia | 2 | 1 | | | 1 | |
| Senile Dementia | | 2 | 1 | | | |
| Stuporous Insanity | | 1 | | | | |
| Terminal Dementia | 1 | 2 | 1 | | 1 | |
| Typhomania | | 1 | | | | |
| Totals | 15 | 20 | 2 | 2 | 3 | 4 |

Total males, 20; females, 26.

Of those admitted during the year thus, 10 per cent. died in that year, and 25 per cent. were discharged, of whom one-fifth probably permanently recovered, the other 20 per cent. may return to this or some other asylum or remain at home unrecovered, but out of the 65 per cent who remained to swell the resident list, more will die or be discharged in succeeding years, so our estimates concern the events of this year alone, and have nothing to do with ultimate results.

The immediate cause of death of the insane is often difficult if not impossible to discover, and the omnibus "exhaustion" from the psychosis is unavoidably used in assigning a cause for death.

Cardiac failure, either through trophic changes in the heart itself or the pneumogastrics, is a common cause of death among the insane. Gradual or sudden dyspnœa or œdema ending in anasarca are prodromata of final illnesses.

Phthisis pulmonalis ended two melancholiacs, one of whom was a lactational case. It also was the cause of death in one case of mania in puerpero, and in three cases of simple mania. I found fatty degeneration of the heart in post-mortem examination of one paranoiac, one epileptic and one simple maniac.

One case of mania, which had made a good recovery from the mental trouble, died from typhoid pneumonia pending his discharge. That disease cut short the career of one paretic, and another of the same psychosis died of the pneumonic invasion which has been noted by alienists as so common an accompaniment of that form of insanity.

Anasarca concluded one case of hebephrenia, one of senile dementia, one of melancholia and one of epilepsy. One paretic dement died from facial erysipelas. Dysentery was the final sickness of one melancholiac and one alcoholic insane. Hemiplegia and stupor preceded the death of one senile dement, a condition always imminent where degenerate, as atheromatous, arteries exist. The same disorder was the forerunner of death in one paretic dement. One maniac was paraplegic just before he died. Two female melancholiacs emaciated to an extreme and gradually passed away from the effects of innutrition. One epileptic insane had a large steatoma in his brain, as reported in May last. He is the one mentioned as having died from fatty degeneration of the heart.

One maniac died from the rupture of a hæmatoma in the dura near the superior longitudinal sinus into the left paracentral gyrus of the cerebrum.

The brains of the typhomaniacs were dropsical and difficult to preserve for microscopical examination. Those of terminal dements were uniformly shrunken and with decided increase of the outer neuroglia layer, with atrophy of the nerve cells. In some the convolutions were shrunken and the fissures gaped apart, especially upon the left side of the brain. One case of imbecility in paretic dementia in a female had in addition to the usual lesions of paresis a warped cerebellum and distorted medulla. The left cerebrum was under weight in every case examined.

In chronic cases, where there has been exaltation to a great degree, extravasations from the smaller arteries were abundant, particularly along both sides of the superior longitudinal sinus in the exterior dura.

In a few cases hæmatoma auris was found as noted in reports of individuals, and always it was the left ear affected.

Female Case No. 341, now living here, is the only case of right hæmatoma auris I have seen, and the other ear subsequently became similarly affected, making it double. One small arachnoid cyst with serous contents was found in the cerebellum of a simple melancholiac, and a blood cyst in the base of the cerebrum of one secondary confusional from mania.

The melancholiac brains have been found to be very pale, but otherwise as not exhibiting any remarkable change from normal. Most cases of melancholia seem due to depressed blood conditions and the agitated forms toxæmic. One case of melancholia agitata was accompanied with Bright's disease, and I ascribe the mental condition to the toxic circulatory, notwithstanding the comparative rarity of such cases.

Cerebral pathologists are familiar with the leucocytic exudate into the arachnoid which covers the brain with a film of a creamy color. This, with thickening of the leptomeninges, I have noted in most cases of mania and its terminal conditions. In typhomania this is excessive.

As to the causes of the different psychoses, I have too many notes to be able to utilize them in this article. Predisposition has very much to do with the form the insanity will assume under similar causes. For instance, traumatism may cause dementia, melancholia, mania or paranoia, according to the inherited disposition of the individual and the nature of the lesion. Frontal head injuries accompany, if they have not directly caused, many cases of paranoia without other apparent cause. Slight moral causes often precipitate paranoia where there was an inherited disposition toward mental weakness. Alcoholism preceded many cases of simple mania and a few cases of primary confusional, and it is an undoubted cause of the alcoholic form, without any other factor operative, though it is conceivable that an inherited predisposition to insanity would require less of the poison to establish it. Fright will cause epilepsy in one and melancholia in another. Katatonia seems to have its origin in the motor apparatus, and I incline to regard the stagy behavior and hallucinations of voices commanding rigidity as suggested to the mind by the forced attitudes, precisely as an excellent digestion and circulation suggests exhilerant ideas and causes one to see everything *couleur de rose.* I have found urea secreted in abnormal quantity from two cases in the cataleptoidal stage.

One case of katatonia followed typhoid fever; another a strumous condition; one was sunstruck before becoming katatoniac; one male and one female case were "peculiar" mentally from birth, the male upon admission showing phases of hebephrenia. I have noticed some of these patients complaining in the beginning of rheumatic stiffness of the joints before catalepsy and delusions appeared. Katatonia seems to be allied ætiologically to some muscular rheumatoid disease, though its origin may be in nerves or blood. As showing what predisposition will do toward changing the nature of a psychosis, Mary R., Case No. 307, would have been ordinarily a case of melancholia ex lactatione, but was a decided paranoiac with depressing delusions of persecution.

I sent a report of 105 MS. pages to the American Neurological Society, June 17, meeting in New York, embodying some researches I have made upon the vaso motor system, with pathological applications and suggestions concerning the treatment of certain forms of insanity. The paper, entitled "Nervous and Mental Physics," will appear in the August, 1884, *American Journal of Neurology and Psychiatry.* The paper is much too lengthy to be fairly abstracted here, but something of its tenor may be noted in the following concluding words:

One of the most important practical deductions that can be made from a recognition of the full identity of nervous and mental processes, in my opinion, is the necessity for regarding *psychic pain and kindred aberrations as calling for treatment continuous and persistent,* based upon rational therapeutics. Melancholia, mania, etc., are but symptoms of grave bodily disease, and precisely as in peritonitis pain is a guiding symptom for therapeutic succor, there is just as great a demand for constant antagonism of destructive cerebral disease, of which psychic pain is as symptomatic as general pain is in peritonitis and rheumatism of other organic disturbances. Ergot, conium, opium, stimulants, etc., with *intelligent and humane* care of the insane, are powerful aids toward bridging over the danger which threatens the mind. The cyclical nature of many forms of insanity are as well marked as is the self-limitation of typhoid fever; but where resolution and reorganization of hepatic or pulmonary tissues need not endanger life, that of the brain tissue leaves its mark for all time upon the psychic life. Hence the invasion should be fought from the outset so as to limit the necessity for reorganization (which is often cicatricial) as far as possible. Every step of insanity should be contested. Sleeplessness, furor, convulsions, overwhelming grief or excitement should be allayed constantly and judiciously with the powerful therapeutic agents at our disposal. Even in so hopeless a psychosis as that of paretic dementia, I have known remissions to be prolonged by such treatment, and for the time being the patient was capable of making provision for the future of his family.

If we are ever to be able to conquer this terrible malady, it will be through adopting some such measures as those mentioned, and never through the prevailing *laisser faire.*

